# Dr. John P. Fleming

**Published:** 1869-02

**Authors:** 


Dr. John P. Fleming.—We are under the painful necessity of chronicling the death of our old and much esteemed friend, John P. Fleming, M. D., of Baltimore, Maryland. The Doctor died Aug. 13th, in the 40th year of his age, after a very brief illness. He was a man of high professional worth, beloved by many friends, a kind husband and devoted christian. May God bless and protect the widow and little ones, whose cheeks have been furrowed by the tears of sadness.

